# Expression of the Inherently Autoreactive Idiotope 9G4 on Autoantibodies to Citrullinated Peptides and on Rheumatoid Factors in Patients with Early and Established Rheumatoid Arthritis

**DOI:** 10.1371/journal.pone.0107513

**Published:** 2014-09-15

**Authors:** Geraldine Cambridge, Rita A. Moura, Tania Santos, Akif A. Khawaja, Joaquim Polido-Pereira, Helena Canhão, Maria J. Leandro, João E. Fonseca

**Affiliations:** 1 Centre for Rheumatology, University College London, London, United Kingdom; 2 Rheumatology Research Unit, Instituto de Medicina Molecular, Faculdade de Medicina da Universidade de Lisboa, Lisbon, Portugal; 3 Centre for Inflammation and Tissue Repair, University College London, London, United Kingdom; 4 Rheumatology Department, Centro Hospitalar de Lisboa Norte, EPE, Hospital de Santa Maria, Lisbon, Portugal; Institute of Immunology, Rikshospitalet, Norway

## Abstract

**Background:**

The pre-symptomatic stage of Rheumatoid arthritis (RA) is associated with pro-inflammatory cytokines and autoantibodies. High levels and epitope spread by Rheumatoid factors (RhF) and autoantibodies to citrullinated proteins signify progression towards disease expression. In established RA, the persistence of high autoantibody levels reflects production by both long-lived plasma cells and short-lived plasmablasts. Neither the relative contributions to pathogenesis by autoantibodies from either source, nor the factors responsible for deciding the fate of autoantigen specific ‘parent’ B-cells, is understood. Phenotypic markers identifying subsets of autoreactive B-cells are therefore of interest in understanding the origin and perpetuation of the autoimmune response in RA. One such phenotypic marker is the rat monoclonal antibody, 9G4, which recognises an idiotope on immunoglobuins derived from the inherently autoreactive VH-gene, VH4-34. We therefore investigated whether the 9G4 idiotope was expressed on autoantibodies in patients with RA.

**Methodology/Principal Findings:**

Sera from 19 patients with established RA and those with <1year history of untreated polyarthritis either resolving into RA (n = 42) or non-RA diagnosis (n = 31) were included. Autoantibodies to cyclic citrullinated peptides (CCP), RhF and co-expression of the 9G4 idiotope were measured by ELISA. 9G4 recognised a population of anti-CCP antibodies in the majority of sera from patients with established disease and also in samples from patients with early disaese. 9G4+RhF levels were generally lower and not associated with positivity for, or levels of 9G4+CCP.

**Conclusions/Significance:**

The persistence of 9G4+ immunoglobulins, of any isotype, in serum is rare. We describe here the novel finding of 9G4 expression on anti-CCP antibodies in patients from the earliest symptoms of RA through to established disease. Our results suggest that 9G4 expression on anti-CCP autoantibodies was not due to polyclonal expansion of VH4-34-encoded immunoglobulins. These studies may therefore provide a new focus for investigation into the evolution of the autoimmune response in RA patients.

## Introduction

The serology of patients with Rheumatoid arthritis (RA) is characterised by persistently raised levels of autoantibodies of two main specificities, being those against Fc of IgG (Rheumatoid factors, RhF) and to peptide sequences on a number of different proteins which have undergone citrullination (anti-citrullinated protein/peptide antibodies - ACPA) [1]–[3]. Multiple isotypes of both RhF and ACPA and epitope spread of ACPA can precede the development of clinical disease by many months or years [4]–[8]. Detection of ACPA in the clinical setting however is usually through binding to commercially prepared combinations of cyclic citrullinated peptides (CCP), which detect antibodies of most but not all specificities [9]. The RhF response can also be both exaggerated and show evidence of somatic hypermutation [10], [11]. Although unaffected relatives and relatives with undifferentiated arthritis can also have ACPA and RhF, albeit at much lower titres, the number of isotypes is more limited and sera from patients with undifferentiated arthritis also recognise fewer citrullinated epitopes [12].

The usage of some genes encoding particular variable heavy chains of immunoglobulins (IGVH) has been associated with the development of autoantibodies [11], [13], [14], with those encoded by VH4-34 being the prototype [15]. Identification of B-cells and antibodies using this VH gene is possible using the rat monoclonal antibody, 9G4, which recognises a unique comformational epitope largely confined within framework 1 of VH4-34-derived immunoglobulins (Igs) [16]. This epitope is inherently autoreactive as it recognises N-acetyllactosamine (NAL) on a number of microbial glycolipids, self glycoproteins and on cells undergoing apoptosis [17]–[19]. 9G4+ B-cells can constitute up to 10% of peripheral B-cells although VH4-34-derived serum Igs constitute less than 1% of total Igs [20]. The level of VH4-34-derived Igs can however transiently increase in response to infections [19], [21]–[23]. The ability to recognise a number of self-antigens via the NAL-epitope may thus be advantageous in clearing damaged, apoptotic or neoplastic cells, but may also increase the risk of autoimmunity, particularly if the conventional binding site on the same molecule undergoes mutation to an autoreactive specificity. VH4-34 gene usage has been shown to be obligatory for the production of most pathogenic IgM cold-agglutinins and has been for demonstrated in IgM-RhF, IgG anti-dsDNA antibodies in systemic lupus erythematosus (SLE), and IgM anti-myeloperoxidase antibodies in systemic vasculitis [24]–[27]. The consequence of allowing the inherently autoreactive VH4-34-derived B cell populations to persist within the B-cell repertoire, across all ethic groups, implies that differentiation to antibody secretion, except in the context of infection, must be under strict physiological control. Censoring of 9G4+ B-cells may be due to anergy associated with high-dose antigen exposure, as the apparent block to maturation into Ig secretion can be overcome by culturing isolated 9G4+ cells *in vitro* with cytokines and CD70 stimulation [22].

Preliminary studies in our laboratory showed that sera from some patients with established RA contained 9G4+ antibodies to CCP [28]. In the present cross-sectional study, we have extended analysis of 9G4 expression to include sera from patients with the earliest symptoms of RA (<6 weeks polyarthritis) and disease controls, and in patients with established disease, using samples from a well-described cohort [29]. We have also measured serum autoantibody levels and class and included anti-microbial antibodies in the analysis.

## Patients and Methods

### Patients

Patients were attending the Rheumatology Department in the Hospital de Santa Maria, Lisbon Academic Medical Centre and Hospital da Luz, Lisbon, Portugal. Serum samples were collected from 40 consecutive patients with untreated polyarthritis of less than 6 weeks duration of symptoms and from 33 untreated early polyarthritis patients with >6 weeks and <1 year of disease history. Serum samples were also collected from 19 patients with established seropositive RA from Lisbon Academic Medical Centre (Est-RA) Samples from Lisbon centers were stored at Biobanco-IMM, Lisbon Academic Medical Centre. All patients with established disease had received a variety of therapies but patients currently or previously treated with rituximab were excluded. Serum samples from 21 healthy controls (HC) were also collected. The local ethics committees (Comissão de Ética do Hospital de Santa Maria, Lisbon, Portugal and University College London Ethics Committee, London, United Kingdom) approved the study and all patients signed an informed consent prior to any protocol-specific procedure.

### Measurement of IgM and IgA Rheumatoid factor (RhF)

RhF were detected using affinity purified rabbit IgG (Sigma Aldrich, St Louis, USA) as substrate. Binding of sera (diluted 1/200) on coated and uncoated wells was measured using goat-anti-human IgA- or IgM-horeradish peroxidase (HRP) conjugate (The Binding Site, Birmingham, UK). In all ELISAs, color development was with tetramethylbenzidine (TMB-Sigma-Aldrich, Gillingham, UK). After subtracting background binding (to uncoated wells), arbitrary units (AU) of binding were calculated by reference to control sera and a standard curve constructed from high positive control sera for each isotype. Cuts-off were 10 AU/ml and 23 AU/ml for IgM and IgA-RhF respectively. IgG-RhF was detected following pepsin digestion of sera with detection of IgG-Fab_2_ fragments using HRP-conjugated goat anti-IgG-Fab_2_, as previously described [30]. Reagents were supplied by TheraTest (Chicago, USA).

### Anti-cyclic citrullinated peptide antibodies

Antibodies to citrullinated peptides were measured using commercial ELISA plates coated with second generation citrullinated peptides (CCP2) (FCCP600; Axis Sheild Diagnostics, Dundee, UK). Sera was added to plates at a dilution of 1/200. HRP-conjugated sheep anti-human IgG, −IgM or –IgA (The Binding Site, UK) were used to detect classes of anti-CCP2, and the assay was developed as above for RhF. Levels of IgG-CCP2 were calculated as per manufacturers instructions and IgM- and IgA-CCP2 calculated following in-house protocol. Cut-off values were defined according to the manufacturer’s instructions for IgG-CCP2 (5 U/ml) or defined as mean ± 3SD from healthy controls for IgM-CCP2 and IgA-CCP2 (26 AU/mL and 10 AU/mL, respectively).

### Expression of 9G4 on RhF and on antibodies to CCP2

For detection of 9G4 expression on either RhF or CCP2, sera (diluted 1/50 in RD6Q diluent, R & D Systems, Abingdon, UK) were added to respective antigen coated wells of ELISA plates. Following initial incubation, the 9G4 reagent (IGM Bioscience, Palo Alto, USA) was added at a concentration of 2 µg/ml to one side of the plate, the duplicate serum-incubated wells receiving diluent buffer instead of 9G4. 9G4 binding to RhF was detected using biotinylated goat anti-rat IgG (Abcam, Cambridge, UK), and a streptavidin–HRP visualization system. An affinity purified HRP-conjugated sheep anti-rat-IgG reagent (Amersham, UK) was used to detect 9G4 recognition of CCP2-binding antibodies. Results were calculated and presented as optical densities (OD at 450 nm) following the subtraction of any background binding in the absence of the 9G4 reagent. Results exceeding mean ± 3 SD of 5 HC sera were regarded as positive.

### Separation of sera using Protein G columns

Sera from 4 patients with established RA which had both IgM- and IgG-class autoantibodies to CCP2, which also bound the 9G4 reagent, were separated on Protein G columns using conventional techniques [31]. Eluted fractions (using low-pH) and flow-through fractions were then tested for IgM-, IgG- and 9G4-CCP2 activity, as described above.

### Statistical analysis

Statistical differences were determined using GraphPad Prism (GraphPad, San Diego, USA). For populations not following a normal distribution, non-parametric statistics were used (Mann-Whitney U test) to compare independent groups. Linear regression (Spearman’s rank correlation) was used to determine relationships between variables. Differences were considered statistically significant for *p*<0.05.

## Results

### Characteristics of patient cohorts

Clinical details of patients with established RA, untreated polyarthritis, together with HC, are shown in Table 1. In patients with a history of polyathritis of <6 weeks (n = 40), after a minimum follow-up of 3 months, 14/40 patients fulfilled the 2010 American College of Rheumatology (ACR)/European League Against Rheumatism (EULAR) criteria for RA [32] and were classified as Very Early Rheumatoid Arthritis (VERA) patients. The remaining early polyarthritis patients (n = 26), who did not evolve into RA, were classified as Very Early Non-RA (VENRA). Patients in the VENRA group were resolved into diagnostic categories of spondyloarthritis (6), SLE (5), polymyalgia rheumatica (4), microcrystalline arthritis (2), Sjögren syndrome (1), undifferentiated diffuse connective tissue disease (1), viral polyarthritis (1), arthritis associated with malignancy (1) and 5 patients entered spontaneously into remission (self-limiting polyarthritis). In the second group of untreated polyarthritis patients (n = 33), who had more than 6 weeks history but less than one year of disease duration, those who after a minimum follow-up of 2 years fulfilled the 2010 ACR/EULAR criteria for RA (n = 28) were classified as Early RA (ERA) [32]. The remaining 5 untreated early polyarthritis patients who did not evolve into RA were classified as Early Non-RA (ENRA) (all with undifferentiated arthritis). Comparing VERA with VENRA and ERA with ENRA, there were no significant differences with respect to age, number of tender joints, ESR or DAS28, although patients in the ERA cohort had significantly more swollen joints than ENRA (Table 1).

**Table 1:** Demographic data of healthy controls, patients with untreated polyarthritis and patients with established RA.

Clinical features	Healthy Controls	VERA	VENRA	ERA	ENRA	Est-RA
*Median and range*						
*unless otherwise indicated*	**(n = 21)**	**(n = 14)**	**(n = 26)**	**(n = 28)**	**(n = 5)**	**(n = 19)**
Age (years)	52 (22–66)	50 (26–94)	41 (24–83)	48 (17–79)	45 (41–66)	54 (21–77)
Sex (% female)	67	93	73	82	80	68
Disease duration	NA	<6	<6	8 (2–12)	3 (3–11)	5 (1–20)
*(time)*		weeks	weeks	months	months	years
Swollen joints	NA	16 (0–28)	2 (0–22)	6 (0–26)*	1 (0–7)*	3 (1–22)
Tender joints	NA	12 (1–28)	7 (0–25)	6 (0–26)	3 (1u7–7)	4 (1–22)
ESR (mm/1^st^ hour)	ND	39 (3–95)	27 (4–120)	21 (2–120)	9 (6–33)	25 (5–95)
DAS28 (mean ± SD)	NA	6.1±1.7	4.9±1.9	5.1±1.4	3.8±1.3	4.7±1.4

NA^a^: not applicable, ND^b^: not determined, *p<0.05 (Mann Whiney Rank sum).

Results for healthy controls (HC) and patients with untreated polyarthritis of either ≤6 weeks duration (Very Early-VE) and subsequently diagnosed as RA (VERA) or Non-RA (VENRA) and patients with >6 weeks and ≤1 year history of polyarthritis (Early-E) subsquently diagnosed with RA (ERA) or a different clinical diagnosis Non-RA (ENRA). Est-RA: Established RA.

### 9G4+autoantibodies in early and established RA

**Figure 1:**
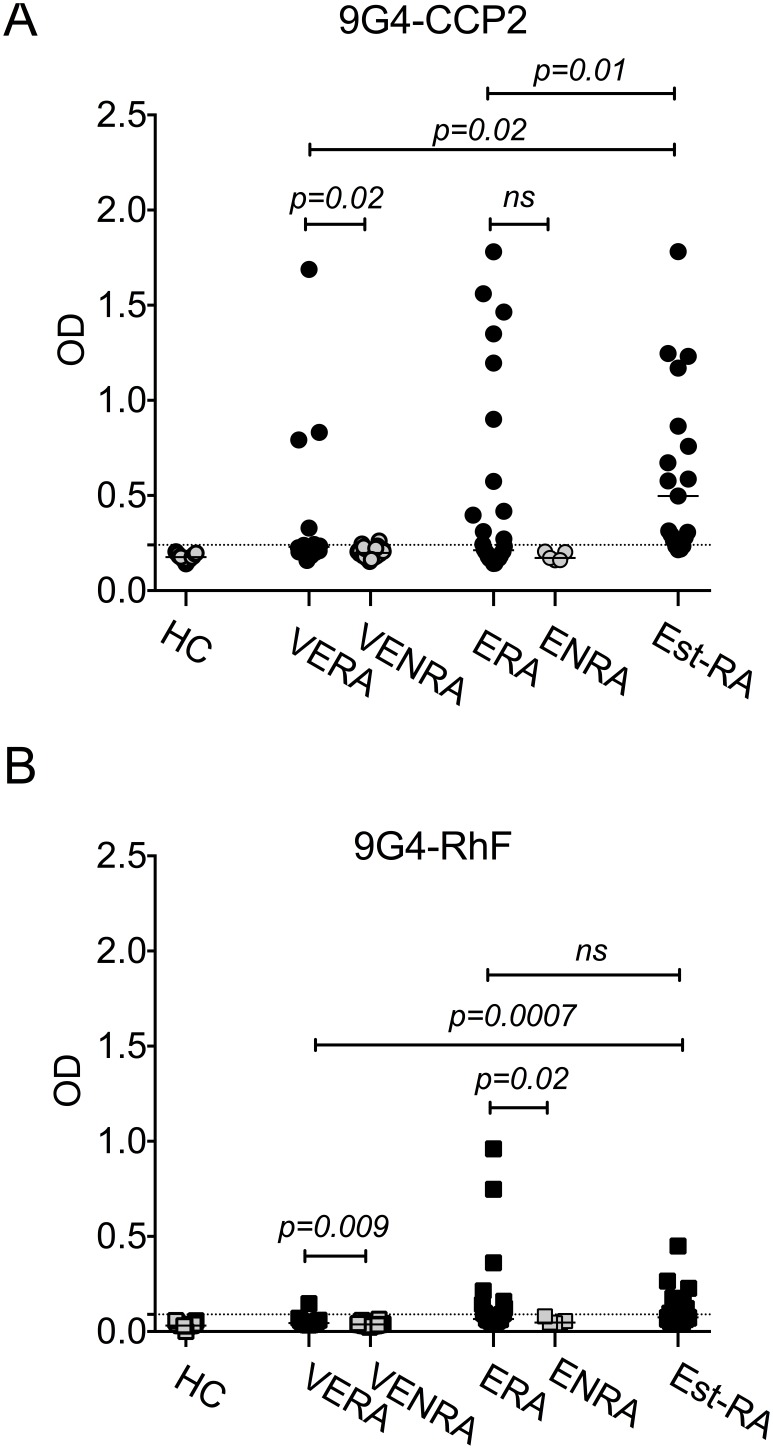
9G4 expresssion on antibodies to CCP2 and on RhF in early and established RA. 9G4 expression on CCP2 (A) and RhF (B) in sera from healthy controls (HC), patients with very early RA (VERA), very early non-RA (VENRA), early RA (ERA), early non-RA (ENRA) and a cohort of seropositive established RA (Est-RA). Comparisons were with Mann Whitney U test at 5% level.

**Figure 2:**
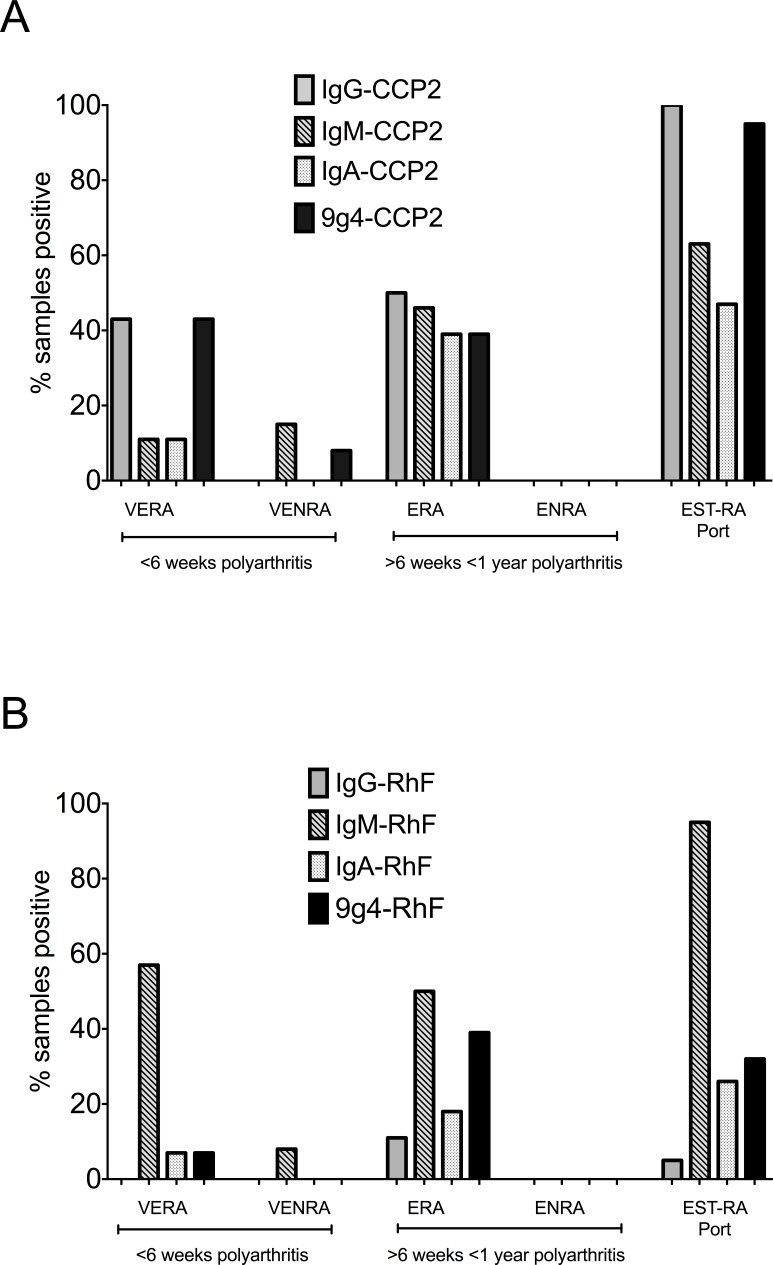
Percentages of samples positive for IgG-, IgA- and IgM- anti-CCP2 antibodies and RhF and for 9G4 expression. Isotypes of anti-CCP2 antibodies and 9G4 expression on CCP2 binding antibodies are shown in (A) and isotypes of RhF and 9G4 expression of antibodies binding to rabbit-IgG are shown in (B).

Although the numbers of patients in each early RA cohort were low, relative levels of 9G4 binding to each autoantigen specificity in patients and HC showed that overall, 9G4 binding to the citrullinated substrate appeared greater than that by RhF (Figure 1A and B). 9G4 expression on autoantibodies in VERA patients was largely restricted to those targeting citrullinated peptides (6/14 compared with 1/14 for RhF). 9G4 expression was borderline positive on CCP2-binding antibodies in 2 samples in the VENRA control group. In the ERA cohort, 9G4 expression was similar for both autoantibody specificities but in Est-RA, virtually all sera contained 9G4+CCP2 activity (18/19) compared with 6/19 positive for 9G4+RhF. In Figure 2A and B, IgG-CCP2 and IgM-RhF were the main specificities present in patients with the earliest disease (VERA cohort). IgM class autoantibodies of both specificities, but not IgA- or IgG- autoantibodies, were also present in a proportion of VENRA patients (4/26 with IgM-CCP2 and 2/26 with IgM-RhF), but not in patients in the ENRA cohort (0/5). 9G4+ expression was borderline positive on CCP2-binding antibodies in 2 samples in the VENRA control group, both of which contained IgM-CCP2 only (Figure 2A). The percentage of samples positive for 9G4+CCP2 in the ERA cohort was the same as that for 9G4+RhF (39%). The percentage of samples positive for 9G4 binding in the established RA cohort however was nearly 100% whereas the percentage containing 9G4+RhF remained around 30%. Levels of binding also appeared considerably lower for 9G4+RhF than for 9G4+CCP2 (Figure 2B; Figure 1).

### Comparison of isotypes and 9G4 expression in individual patients

**Figure 3:**
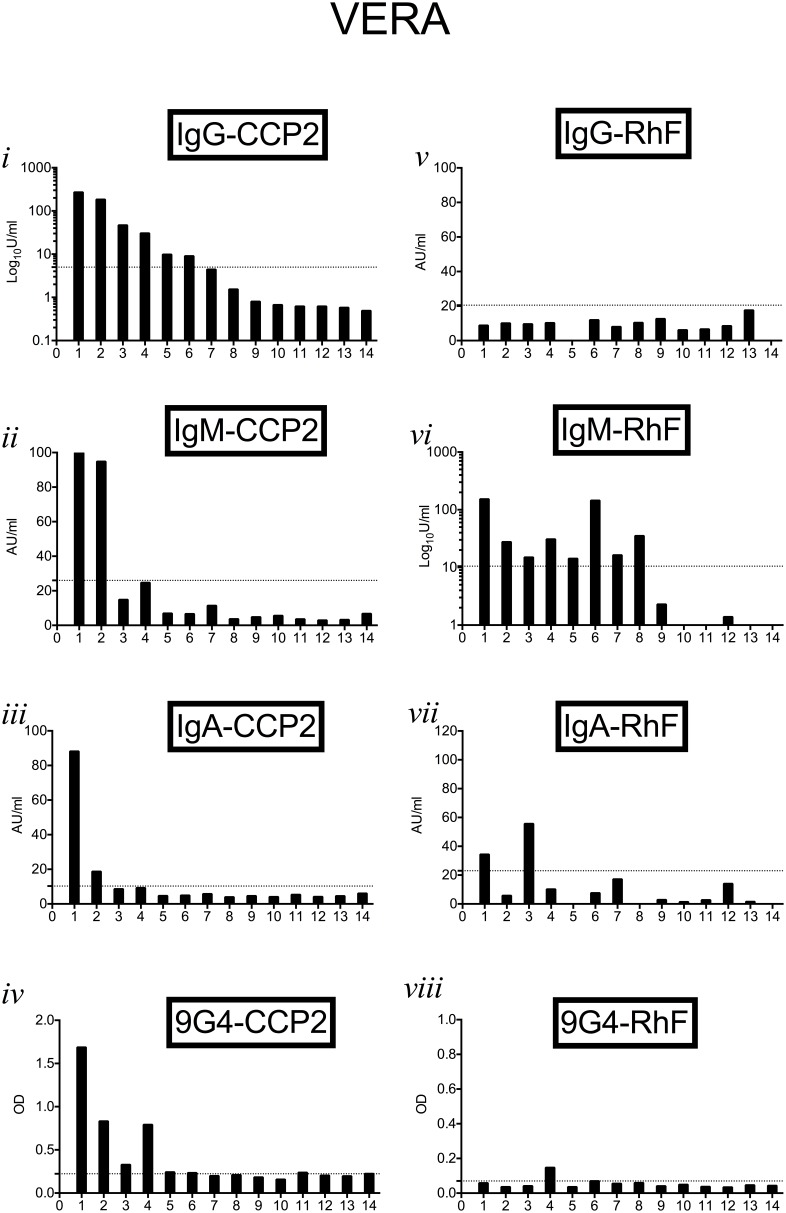
9G4 expression and relative levels of autoantibodies in individual patients in the ERA cohort. Results for all autoantibody isotypes and for 9G4 expression were ranked in order of the relative levels of IgG-CCP2 in order to follow results for individual patients (as numbered on x-axis). Horizontal dotted lines show upper limit of normal ranges determined for each parameter as described in the text.

**Figure 4:**
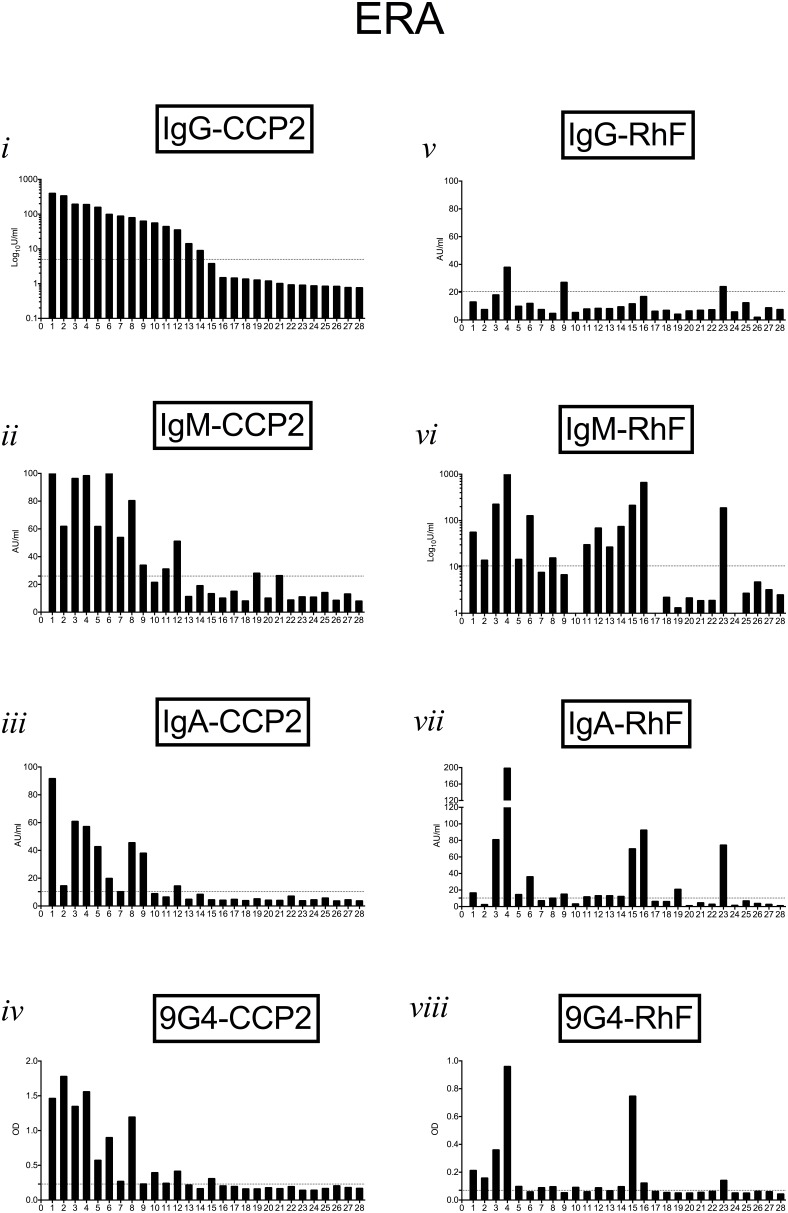
9G4 expression and relative levels of autoantibodies in individual patients in the ERA cohort. Results for all autoantibody isotypes and for 9G4 expression were ranked in order of the relative levels of IgG-CCP2 in order to follow results for individual patients (as numbered on x-axis). Horizontal dotted lines show upper limit of normal ranges determined for each parameter as described in the text.

**Figure 5:**
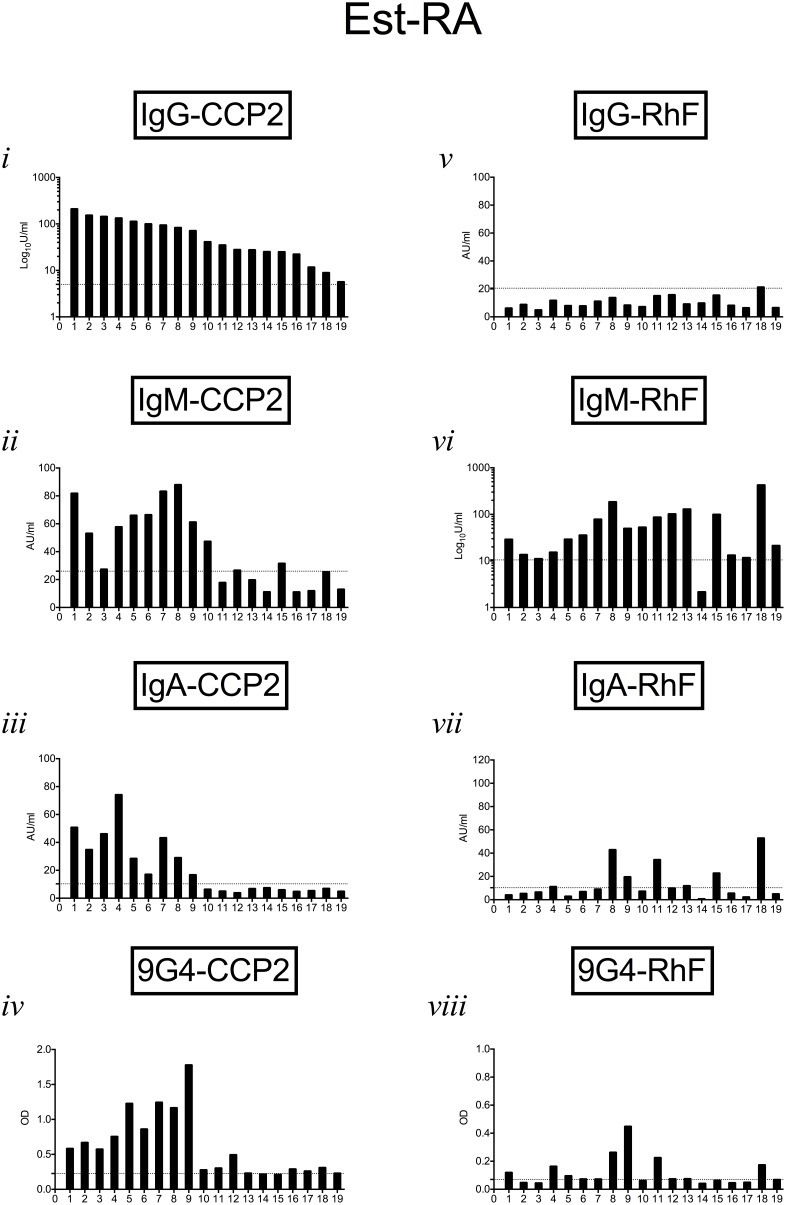
9G4 expression and relative levels of autoantibodies in individual patients in the Est-RA cohort. Results for all autoantibody isotypes and for 9G4 expression were ranked in order of the relative levels of IgG-CCP2 in order to follow results for individual patients (as numbered on x-axis). Horizontal dotted lines show upper limit of normal ranges determined for each parameter as described in the text.

Figures 3–5 show autoantibody isotypes (anti-CCP2 and RhF) and corresponding 9G4 expression in sera from individual patients in the VERA, ERA and Est-RA cohorts respectively. Serum autoantibody results were ranked on the basis of the relative levels of IgG-CCP2 antibodies in order to compare positivity for individual specificities in each patient. In the VERA cohort (Figure 3), 9G4 expression on CCP2-binding antibodies was evident in all samples which also had significant IgG-CCP2, although 2 had very low/borderline expression. IgM- and IgA-CCP2 were present in only 2 of these samples. IgM-RhF was present in 8 samples, 2 of which were also positive for IgA-RhF, with only 1 sample being 9G4+RhF. Figure 4 similarly shows results for patients within the ERA group. IgG-CCP2 positivity was usually accompanied in the same sample by IgM-CCP2, IgA-CCP2 and 9G4+CCP2. The presence and level of the RhF response and 9G4+RhF expression did not correlate with presence or levels of anti-CCP2 antibodies in the same patient. In the Est-RA cohort (Figure 5), samples with the highest levels of IgG-CCP2 also contained IgM- and IgA-CCP2 and most were also positive for IgM-RhF. The pattern of 9G4+CCP2 positivity corresponded most closely with that of samples containing IgM- and IgA-CCP2 as well as IgG-CCP2.

### Is 9G4 expression on IgM or IgG-anti-CCP2 antibodies?

Protein G columns were used to separate sera from 4 patients with established RA (Figure S1). Following separation, results suggested most 9G4 activity associated with the eluted fraction. However, the eluted fractions contained both IgM- and IgG-CCP2 activity, probably due to interference by Rheumatoid factors. In the flow-through fractions only 1 serum was positive for 9G4-CCP2 binding. This fraction also had CCP2 binding activity in both IgM- and -IgG-CCP2 assays, which may also be due to the presence of high levels of Rheumatoid factors blocking Fcγ binding to Protein G. Results were therefore equivocal but suggested that at least a proportion of 9G4+CCP2 were within the IgG fraction.

## Discussion

The ability to bind the rat monoclonal anti-idiotope antibody, 9G4, was used as a surrogate measure of the utilisation of the VH4-34 heavy chain by the 2 main autoantibody specificities present in sera from patients with RA. Our results showed that in patients with early RA and with established disease, there was a remarkably consistent recognition of anti-CCP2 antibodies by the 9G4 reagent. Positivity for binding of 9G4 to RhF was also found but less frequently. Further, levels of binding to each autoantibody specificity did not correlate in individual patients. This report is the first, to our knowledge, to describe the apparent usage of the inherently autoreactive VH4-34 gene by anti-CCP2 antibodies in patients with RA from the very earliest signs of disease.

Unaffected relatives of RA patients, as well those with an undifferentiated arthritis, can test positive for ACPA (and RhF) [33]. In the present study, we found that although IgM-CCP2 and IgM-RhF could both be present in sera from VENRA and ENRA patients, where synovitis failed to develop into RA, 9G4 expression on anti-CCP2 antibodies was present in only 2 samples, and at very low levels. Both samples were from VENRA patients, and contained only IgM-class anti-CCP2.

In normal individuals, VH4-34 usage by total serum immunoglobulins (both IgM and IgG) is disproportionately low (∼0.7% of serum IgM; no IgG) given that 5–10% of peripheral B-cells express the 9G4 idiotope [20], [34]. Consistent with a lack of class-switched 9G4+Ig, 9G4+ B-cells are absent from tonsillar germinal centres in healthy individuals [35]. Following infections, such as with EBV, antibodies utilising VH4-34 show a transient increase, largely within the IgM pool. Expression of CD27 (expressed by most but not all memory-B-cells [36]) can only be detected on a small (<10%) sub-population of 9G4+ B-cells, but somewhat unusually such cells can co-express the naïve B cell marker, CD5 [37]. Taken together, these observations are consistent with the VH4-34 gene being utilised by naïve or pre-switch B-cells, which display a limited ability to undergo class switch [15]. The presence of such a high proportion of VH4-34-derived B-cells in the naïve compartment further suggest that this particular germ line sequence is preferentially selected for exit from the bone marrow into the periphery, regardless of CDR3 specificity.

In the context of autoimmunity, most notably SLE, class-switched (IgG) 9G4+ anti-dsDNA antibodies are present in serum and in involved renal tissue [25], [34]. 9G4+ B-cells were also abundant in germinal centre (GC) reactions in tonsils from SLE patients but tonsils from RA patients resembled those of normal individuals with 9G4+ B-cells thus being excluded [35]. In another study, VH4-34 derived B-cells from normal human tonsils did show evidence of class switch [38], but the anatomical location of such B cells within the tonsil was not given. Somatic hypermutation was also detected before class switch in some clonally related transcripts, suggesting that VH4-34 (µ+) B-cells were indeed capable of undergoing both somatic mutation and class-switch, most likely outside a GC reaction [35].

We found that in samples from patients with the shortest history of polyarthritis (VERA), 9G4+CCP2 was most strongly associated with IgG-CCP2, particularly when co-existing in samples containing IgM-RhF. Although the relative contribution of different Ig isotypes to total 9G4-CCP2 binding was dificult to interpret due to probable interference with RhF, our results using Protein G columns (Figure S1) and pepsin digestion (data not shown) nevertheless suggested that both IgM- and IgG-CCP2 autoantibodies can express the 9G4+ idiotope, consistent with the possibility that class-switch of 9G4+CCP2 antibodies could occur.

9G4 expression on RhF in the RA cohorts was indeed found to be less common and somewhat lower than on CCP2-directed antibodies. In addition, the levels of 9G4 expression on antibodies to CCP2 and on RhF in individual samples (Figures 3–5) showed that the 9G4 idiotope was not necessarily present on both autoantibody specificities in the same sample and if present, relative expression usually differed. Expansion of 9G4+CCP2 autoreactivity was therefore both greater than that of 9G4+RhF, particularly in early RA patients, and therefore was not likely to be the result of a polyclonal expansion of all ‘autoreactive’ VH4-34-derived parent B-cells. In addition, we found that 9G4 expression was absent from antibodies to tetanus toxoid and pneumococcal capsular polysaccharides within serum samples positive for 9G4-CCP2 (Figure S2). This argues against a prior general expansion of VH4-34 encoded antibodies and further suggests that autoantigen specificity may play a role in the selection of particular 9G4+ B cell clones.

Intriguingly, the percentage of samples positive for IgM- and for IgA-CCP2 between VERA and ERA cohorts followed, rather than preceded positivity for IgG-class antibodies to CCP2 (Figure 2A; Figures 3 and 4). This supports the suggestion that the elicitation of IgG-CCP2 antibodies to a citrullinated protein may arise early in the underlying pathogenic process and also suggest a sequence of events not shared with conventional antibody responses to immunisation or infection [39]. In established RA, the persistence of multiple isotypes of anti-CCP2 over time, most notably in the IgM fraction, also indicates that there is a constant renewal of autoreactive (naïve) B-cells throughout the course of disease which then differentiate into plasmablasts, or long-lived plasma cells [40]. The kinetics of autoantibody production following B-cell depletion treatment with the anti-CD20 reagent, rituximab, have shown that at least a proportion of IgM-autoantibodies derive from early/naïve B-cells continuously exiting the bone marrow; a process interupted by this form of therapy [41]. Support for this scenario comes from the experiments of Samuels et al [42] who reported that VH regions cloned and translated from early naïve B-cells from RA patients contained a high proportion of transcripts with reactivity for citrullinated peptides. This would suggest that in RA, autoreactive B cell specificities escape deletion, receptor editing or anergy early in their development, ultimately giving rise to a population of B-cells which can also survive entry into the mature B cell compartment in the periphery.

The unique properties of B-cells expressing both the inherently autoreactive 9G4 epitope, coupled with specificity and high affinities for citrullinated residues through CDR3 may conceivably therefore be a key factor in the selection and possibly expansion of the immune response to citrullinated proteins in RA patients. Although B cell development in the bone marrow is largely antigen-independent, signaling through a functional B cell receptor complex (consisting of surface immunoglobulin, CD19 and co-receptors CD81, CD21), microenvironmental factors and also interaction between pre-B-cells, influences selection during differentiation towards entry into the peripheral pool. We have previously suggested that the expansion of autoreactive B cell clones in systemic autoimmune diseases relates to pro-survival properties imbued by certain B-cell receptors [43]. VH4-34 usage, combined with specificity for citrullinated residues, may therefore increase the probability of positive BCR signalling in parent B cells and contribute to the selection of auto-reactive B-cells either before their exit from the bone marrow and/or in the periphery of patients with RA.

## Conclusion

In this cross-sectional study, seropositivity for autoantibodies expressing the 9G4 idiotope apparently increased with disease maturity in patients with RA. A higher proportion of patients with the earliest symptoms of polyarthritis whose disease resolved into Rheumatoid arthritis were also more likely to have 9G4+CCP2 than those with other diagnostic outcomes. The presence of 9G4+ antibodies of any isotype in the serum immunoglobulin pool is unusual and is here clearly associated with autoimmunity. 9G4+ autoantibodies were able to be detected in the circulation from the first weeks of resolution into RA and may thus represent a surrogate measure of selection of autoreactive B-cells into the periphery.

## Supporting Information

Figure S1 **9G4 binding to serum fractions separated by Protein G columns.** IgM- and IgG- anti-CCP2 and 9G4+CCP2 reactivity was measured in sera from 4 patients with established RA. Following separation on Protein G columns, flow through and eluted fractions were tested for IgM-, IgG- and 9G4-CCP2 activity.
(TIFF)

Figure S2 **9G4 binding to antibodies to microbial antigens.** Antibodies to pneumococcal capsular polysaccharides (PCP – consisting of a combination of 23 serotypes of Streptococcus *pneumoniae* serotypes) and to tetanus toxoid (TT) were measured by ELISA using commercial kits. 9G4 expression on bound anti-microbial antibodies were then determined using methods described for autoantibodies.
(TIFF)
