# Supplementary figures and images for: Expression of the Inherently Autoreactive Idiotope 9G4 on Autoantibodies to Citrullinated Peptides and on Rheumatoid Factors in Patients with Early and Established Rheumatoid Arthritis
Source: PLoS One. 2014 Sep 15;9(9):e107513. doi: 10.1371/journal.pone.0107513 (PMC4164660; doi:10.1371/journal.pone.0107513)

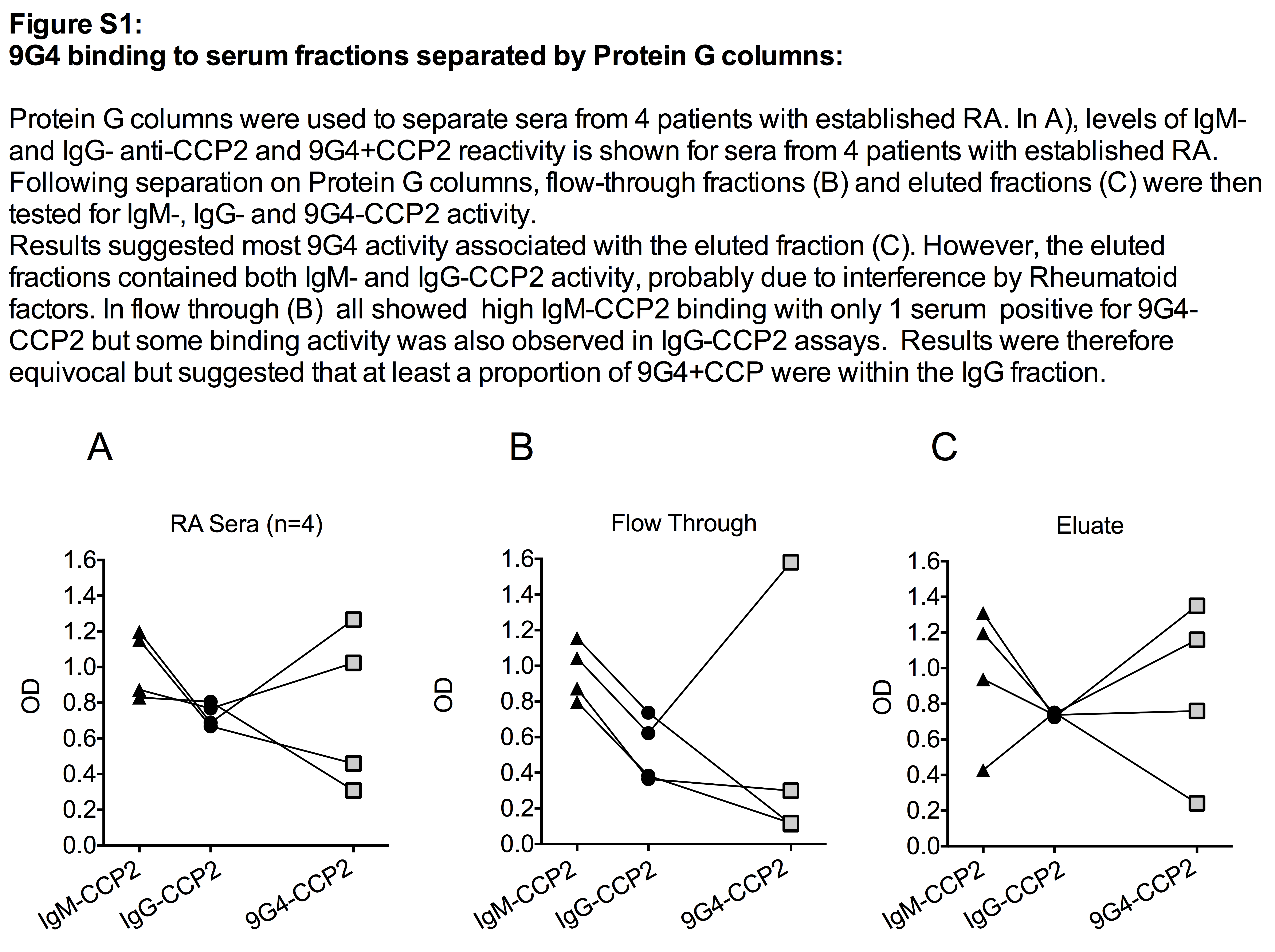

Supplement: Figure S1 — 9G4 binding to serum fractions separated by Protein G columns. IgM- and IgG- anti-CCP2 and 9G4+CCP2 reactivity was measured in sera from 4 patients with established RA. Following separation on Protein G columns, flow through and eluted fractions were tested for IgM-, IgG- and 9G4-CCP2 activity. (TIFF) [file pone.0107513.s001.tiff]

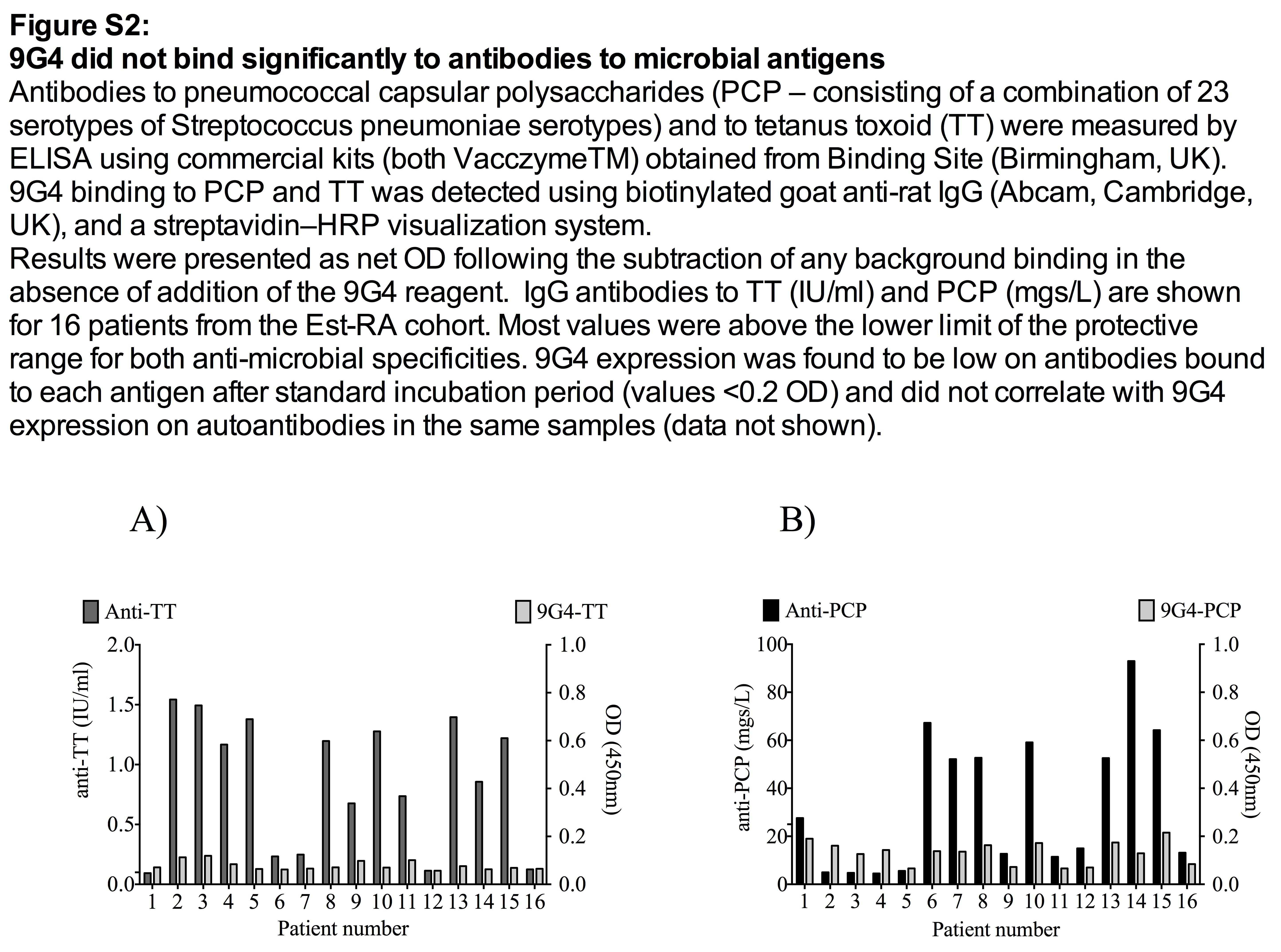

Supplement: Figure S2 — 9G4 binding to antibodies to microbial antigens. Antibodies to pneumococcal capsular polysaccharides (PCP – consisting of a combination of 23 serotypes of Streptococcus pneumoniae serotypes) and to tetanus toxoid (TT) were measured by ELISA using commercial kits. 9G4 expression on bound anti-microbial antibodies were then determined using methods described for autoantibodies. (TIFF) [file pone.0107513.s002.tiff]
